# Evaluating the antimicrobial efficacy of ceftriaxone regimens: 1 g twice daily versus 2 g once daily in a murine model of *Streptococcus pneumoniae* pneumonia

**DOI:** 10.1093/jacamr/dlae092

**Published:** 2024-06-04

**Authors:** Hideo Kato, Mao Hagihara, Shun-Ichi Hiramatsu, Hiroyuki Suematsu, Naoya Nishiyama, Nobuhiro Asai, Hiroshige Mikamo, Kazuko Yamamoto, Takuya Iwamoto

**Affiliations:** Department of Pharmacy, Mie University Hospital, Mie, Japan; Department of Clinical Pharmaceutics, Division of Clinical Medical Science, Mie University Graduate School of Medicine, Mie, Japan; Department of Clinical Infectious Diseases, Aichi Medical University, Aichi, Japan; Department of Clinical Infectious Diseases, Aichi Medical University, Aichi, Japan; Department of Molecular Epidemiology and Biomedical Sciences, Aichi Medical University Hospital, Aichi, Japan; Department of Pharmacy, Mie University Hospital, Mie, Japan; Department of Clinical Infectious Diseases, Aichi Medical University, Aichi, Japan; Department of Infection Control, University of the Ryukyus Hospital, Okinawa, Japan; First Department of Internal Medicine, Division of Infectious, Respiratory, and Digestive Medicine, University of the Ryukyus Graduate School of Medicine, Okinawa, Japan; Department of Clinical Infectious Diseases, Aichi Medical University, Aichi, Japan; Department of Clinical Infectious Diseases, Aichi Medical University, Aichi, Japan; Department of Infection Control, University of the Ryukyus Hospital, Okinawa, Japan; First Department of Internal Medicine, Division of Infectious, Respiratory, and Digestive Medicine, University of the Ryukyus Graduate School of Medicine, Okinawa, Japan; Department of Pharmacy, Mie University Hospital, Mie, Japan; Department of Clinical Pharmaceutics, Division of Clinical Medical Science, Mie University Graduate School of Medicine, Mie, Japan

## Abstract

**Background:**

Ceftriaxone is administered in regimens of either 2 g once-daily or 1 g twice-daily for the treatment of pneumonia caused by *Streptococcus pneumoniae*. Previous clinical study suggests the 2 g once-daily regimen is more effective, but comparison of antimicrobial efficacy between are lacking.

**Objectives:**

To assess the antimicrobial efficacy of these two ceftriaxone regimens against *S. pneumoniae* using a murine model of pneumonia.

**Methods:**

The study employed three *S. pneumoniae* isolates with ceftriaxone MICs of 1, 2 and 4 mg/L and two human-simulated regimens based on the blood concentration of ceftriaxone (1 g twice-daily and 2 g once-daily). Antimicrobial activity was quantified based on the change in bacterial counts (Δlog_10_ cfu/lungs) observed in treated mice after 24 h, relative to the control mice at 0 h.

**Results:**

The human-simulated 2 g once-daily regimen of ceftriaxone exhibited significantly higher antimicrobial activity against *S. pneumoniae* isolates with MICs of 1 and 2 mg/L compared with the 1 g twice-daily regimen (1 mg/L, −5.14 ± 0.19 Δlog_10_ cfu/lungs versus −3.47 ± 0.17 Δlog_10_ cfu/lungs, *P* < 0.001; 2 mg/L, −3.41 ± 0.31 Δ log_10_ cfu/lungs versus −2.71 ± 0.37 Δlog_10_ cfu/lungs, *P* = 0.027). No significant difference in antimicrobial activity was observed against the *S. pneumoniae* isolate with a MIC of 4 mg/L between the two regimens (−0.33 ± 0.18 Δlog_10_ cfu/lungs versus −0.42 ± 0.37 Δlog_10_ cfu/lungs, *P* = 0.684).

**Conclusion:**

2 g once-daily regimen of ceftriaxone is more effective for treating pneumonia caused by *S. pneumoniae,* with MICs of ≤2 mg/L.

## Introduction

Community-acquired pneumonia (CAP) represents the world’s most lethal communicable disease.^1^ Mortality rates for CAP exceed 20% in intensive care units.^2^ Furthermore, annual healthcare expenditures for CAP are estimated to be approximately $9501 million in the USA and €5700 million in Europe.^3,4^ Aspiration has been recognized as a common mechanism of CAP, with aspiration pneumonia accounting for 60.1% of CAP cases in Japan.^5^ Thus, the development of optimal therapeutic and management strategies for aspiration pneumonia is crucial for the effective treatment of CAP.

Ceftriaxone, a broad-spectrum, third-generation cephalosporin, is extensively utilized in the treatment of patients with aspiration pneumonia.^6^ The Sanford Guide to Antimicrobial Therapy suggests dosing regimens for ceftriaxone of either 1 g twice daily or 2 g once daily.^7^ In a prior clinical study, we found that the 2 g once daily regimen of ceftriaxone was more effective in clinical and inflammation responses compared with the divided administration (1 g twice daily regimen) in patients with aspiration pneumonia caused by *S. pneumoniae*, while also minimizing adverse events.^8^ Nevertheless, the antimicrobial activities of the two regimens were not compared, as the design of this prior study did not include the analysis of sputum cultures from patients during ceftriaxone therapy.^8^ Therefore, there is a need to provide solid evidence of the antimicrobial efficacy of the 2 g once-daily regimen as an optimal dosing strategy for the treatment of aspiration pneumonia caused by *S. pneumoniae*. We evaluated the antimicrobial efficacy of 1 g twice daily versus 2 g once daily with the murine pneumonia model.

## Materials and methods

### Antimicrobial agents

Commercially available ceftriaxone (purity, >99.9%), provided by Nichi-Iko Pharmaceutical Co., Toyama, Japan, was used for all *in vivo* studies. Ceftriaxone was diluted with normal saline and adjusted based on the average weight of the animals, to achieve the desired concentration immediately prior to each experiment.

### Microorganisms

This study utilized three *S. pneumoniae* isolates (Table 1). These clinically derived pathogens were identified using matrix-assisted laser desorption ionization-time of flight mass spectrometry (MALDI-TOF/MS) equipment (Bruker Biotyper^®^, Bruker Daltonics, Bremen, Germany). The bacteria were preserved at −80°C in skim milk for long-term storage.

**Table 1.** Characteristics of *S. pneumoniae* utilized in this study

Strains	MIC (mg/L)			
	CRO	PEN	LVX	MEM
D-1	1	2	0.5	0.25
A-1	2	2	1	0.5
R-1	4	8	1	1

CRO, ceftriaxone; LVX, levofloxacin; MEM, meropenem; MIC, minimum inhibitory concentration; PEN, penicillin

### Susceptibility test

The minimum inhibitory concentration (MIC) of ceftriaxone was determined using the Etest, in line with the manufacturer’s guidelines (BioMerieux, North America). Additionally, the MICs for various antimicrobials were determined for each isolate utilizing the broth microdilution method, as outlined in the Clinical and Laboratory Standards Institute (CLSI) guidelines.^9^ These determinations were conducted in triplicate to ensure accuracy, and the modal MIC was employed to categorize the isolates.

### Animals

Pathogen-free, 4-week-old Institute of Cancer Research (ICR) mice, each weighing approximately 20 g, were procured from Charles River Laboratories Japan Inc., Yokohama, Japan. The care and housing of these animals were in strict accordance with the recommendations set forth by the National Research Council, ensuring they had access to food and water *ad libitum*.

### Murine pneumonia model

A neutropenic murine pneumonia model, previously described,^10^ was utilized to assess antimicrobial regimens against *S. pneumoniae* in the pulmonary system of mice. Mice were induced into transient neutropenia through intraperitoneal injections of cyclophosphamide at doses of 150 mg/kg and 100 mg/kg of body weight, administered four days and one day prior to bacterial inoculation, respectively. The isolates intended for pulmonary inoculation were initially frozen at −80°C in skim milk. These isolates were then subcultured twice on Trypticase soy agar plates supplemented with 5% sheep blood (Becton, Dickinson & Co., Sparks, MD, USA) and incubated for approximately 24 h at 37°C in at 5% CO_2_. After an 18–24 h incubation of the second transfer, a bacterial suspension of approximately 10^8,9^ colony forming units (cfu)/mL was prepared for inoculation. Final inoculum concentrations were confirmed using serial dilution and plating techniques. Each mouse was administered 0.06 mL of the bacterial suspension intranasally while under anaesthesia, induced with butorphanol (Meiji Seika Pharma Co., Ltd., Tokyo, Japan), medetomidine (ZENOAQ, Fukushima, Japan) and midazolam (Astellas Pharma Inc., Tokyo, Japan). Post-inoculation, mice were assigned at random to either the control group or to specific dosing regimen groups (*n* = 6, respectively).

### Single dose pharmacokinetics of ceftriaxone in infected mice

To study the pharmacokinetics of ceftriaxone in plasma and lung tissue, ceftriaxone was administered as a single-dose monotherapy to neutropenic mice infected with *S. pneumoniae*. Moreover, we simulated humanized doses of ceftriaxone with the area under the concentration-time curve from 0 to 24 h (AUC_0–24_) of blood drug concentration. The mice received subcutaneous injections of ceftriaxone at doses of 200, 400 and 800 mg/kg, each in 0.2 mL of normal saline. The mice were euthanized at 0.25, 0.5, 1, 2, 4, 8, 12 and 24 h after administration of ceftriaxone, respectively, and samples of blood from the axillary artery and lung tissue were collected immediately (*n* = 3 for each time point).

### Instrumentation and chromatographic conditions

Ceftriaxone concentrations were measured at Mie University (Mie, Japan). Concentrations were analysed using high-performance liquid chromatography (HPLC), adhering to a previously published method with modifications.^11^ The HPLC analysis was conducted on a Waters Alliance 2695 HPLC system (Waters, Milford, MA, USA) connected to a Chemcobond 5-ODS-H column (4.0 × 150 mm; ChemcoPlus, Japan) and a Waters 2996 photodiode array detector (Waters, Milford, MA, USA). The column temperature was maintained at 40°C, and the detection wavelength was set at 270 nm. The mobile phase consisted of a 30 mM phosphate buffer (pH 7.0) and methanol mixture (92:8 by volume), with a flow rate of 1 mL/min. The technique’s limit of quantification was established at 2.5 mg/L.

### Antimicrobial activates studies

Two hours following the inoculation of *S. pneumoniae*, mice were administered ceftriaxone (0.2 mL) subcutaneously (*n* = 6 for each treatment group). Control mice received normal saline (0.2 mL) as a placebo. Twenty-four hours post-administration of ceftriaxone, the mice were euthanized via CO_2_ exposure, and their lungs were subsequently harvested. Lungs from each mouse were individually homogenized, and the homogenates underwent serial dilutions with normal saline before being plated on Trypticase soy agar supplemented with 5% sheep blood to determine the bacterial counts in the lungs (cfu/lungs). Additionally, six infected mice were sacrificed just prior to the commencement of ceftriaxone administration to determine the bacterial load in the lungs at 0 h. The antimicrobial activities were quantified based on the detected bacterial counts in the treated group at 24 h and the bacterial counts in the control group at 0 h (Δlog_10_ cfu/lungs).

### Statistical analysis

The evaluation of antimicrobial activity between the regimens was conducted using one-way ANOVA with Bonferroni correction. Statistical analysis was performed using JMP, version 10.0 (SAS, Tokyo, Japan). Differences were deemed statistically significant at *P* < 0.05.

### Ethics

This study was reviewed and approved by the ethics committee of Mie University (No. 2022-3).

## Results

### The MIC of bacterial isolates

Table 1 displays the MICs for ceftriaxone, penicillin, levofloxacin and meropenem. *S. pneumoniae* D-1 was found to be susceptible to all antimicrobials as per CLSI breakpoints.^9^  *S. pneumoniae* A-1 exhibited intermediate susceptibility to ceftriaxone and meropenem. *S. pneumoniae* R-1 was resistant to ceftriaxone, penicillin and meropenem.

### Pharmacokinetics of ceftriaxone

**Figure 1.**
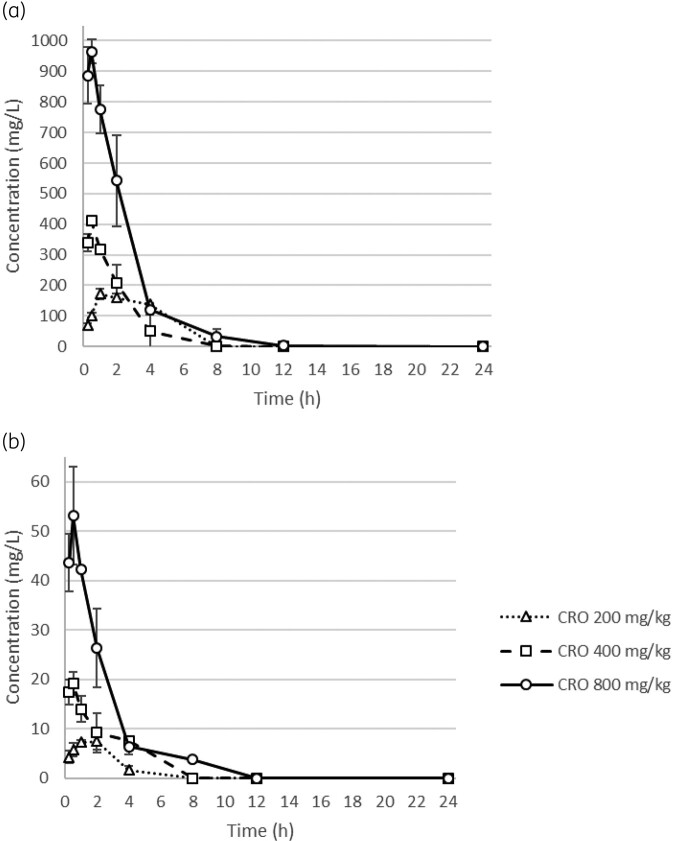
Ceftriaxone concentrations in serum (a) and lung tissue (b) after administration of single subcutaneous doses of 200, 400 and 800 mg/kg to neutropenic infected mice (*n* = 3). Each symbol represents the mean ± SD levels in the plasma. SD, standard deviation.

The pharmacokinetic profile of ceftriaxone following a single subcutaneous dose of 200, 400 and 800 mg/kg is depicted in Figure 1. Peak concentrations (C_max_) in serum and lung tissue were observed at 30 min or 1 h post-administration (serum: 200 mg/kg, 172.16 mg/L [1-h]; 400 mg/kg, 410.07 mg/L [30-min]; 800 mg/kg, 965.11 mg/L [30-min], Figure 1a; lung tissue: 200 mg/kg, 7.25 mg/L [1-h]; 400 mg/kg, 19.18 mg/L [30-min]; 800 mg/kg, 64.45 mg/L [30-min], Figure 1b). The AUC_0–24_ in serum and lung tissue ranged from 838.73 to 2488.12 and 42.47 to 154.69 mg·h/L, respectively. A previous pharmacokinetic study of ceftriaxone in human has reported that the AUC_0–24_ after administration of 1 and 2 g doses were 717.7 and 1571.9 mg·h/L.^12^ Therefore, doses of ceftriaxone equivalent to 236.15 and 520.85 mg/kg for bacterial density studies were employed to simulate humanized doses of 1 and 2 g, respectively.

### Antimicrobial activities against *S. pneumonia*

**Figure 2.**
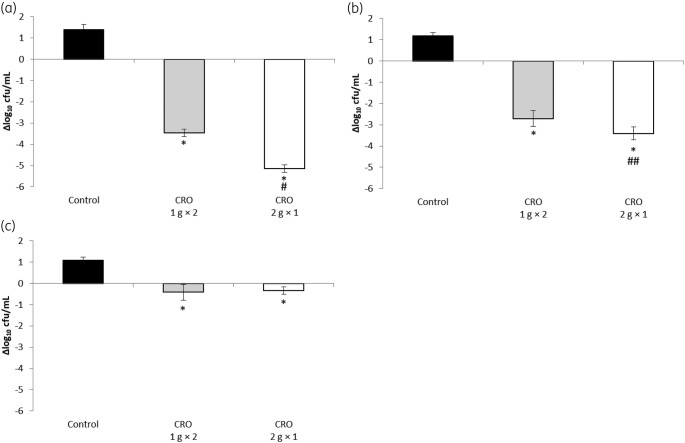
Antimicrobial activity of two dosing regimens (1 g × 2 and 2 g × 1) of ceftriaxone against three *Streptococcus pneumoniae* isolates: (a) D-1 (MIC ceftriaxone 1 mg/L), (b) A-1 (MIC ceftriaxone 2 mg/L) and (c) R-1 (MIC ceftriaxone 4 mg/L) in murine pneumonia model. Change in bacterial counts (Δlog_10_ cfu/lungs) measured in mice after 24 h compared with control mice at 0 h. All data are shown as average ± SD. *P < 0.001 (versus control), #*P* < 0.001 (versus CRO 1 g × 2), ##*P* < 0.05 (versus CRO 1 g × 2). CRO, ceftriaxone; SD, standard deviation.

The results of two regimens of ceftriaxone against three *S. pneumoniae* isolates are shown in Figure 2 (Figure 2a, MIC of 1 mg/L; Figure 2b, MIC of 2 mg/L; Figure 2c, MIC of 4 mg/L).

#### *S. pneumonia* isolate with MIC of 1 mg/L

At the initiation of ceftriaxone therapy (0 h), bacterial loads in the lungs of control mice ranged from 6.42 to 7.24 log_10_ cfu/lungs for *S. pneumoniae* D-1. The growth control exhibited bacterial count increases of 1.13–1.60 Δlog_10_ cfu/lungs after 24 h from the start of ceftriaxone therapy. Ceftriaxone administration significantly reduced bacterial counts of *S. pneumoniae* D-1 compared with those in control animals at 24 h (1 g twice daily regimen, −3.47 ± 0.17 Δlog_10_ cfu/lungs, *P* < 0.001; 2 g once daily regimen, −5.14 ± 0.19 Δlog_10_ cfu/lungs, *P* < 0.001). The 2 g once-daily regimen demonstrated superior antimicrobial activity against *S. pneumoniae* with an MIC of 1 mg/L compared with the 1 g twice-daily regimen (*P* < 0.001).

#### *S. pneumonia* isolate with MIC of 2 mg/L

At the commencement of ceftriaxone therapy (0 h), bacterial loads in the lungs of control mice ranged from 6.82 to 7.23 log_10_ cfu/lungs for *S. pneumoniae* (A-1). The increase in bacterial counts for the control group was between 1.02 and 1.32 Δlog_10_ cfu/lungs after 24 h from the start of ceftriaxone therapy. Ceftriaxone administration resulted in a significant reduction of bacterial counts of *S. pneumoniae* (A-1) compared with those in the control animals at 24 h (1 g twice-daily regimen, −2.71 ± 0.37 Δlog_10_ cfu/lungs, *P* < 0.001; 2 g once-daily regimen, −3.41 ± 0.31 Δlog_10_ cfu/lungs, *P* < 0.001). The 2 g once-daily regimen exhibited superior antimicrobial activity against *S. pneumoniae* with an MIC of 2 mg/L compared with the 1 g twice-daily regimen (*P* = 0.027).

#### *S. pneumonia* isolate with MIC of 4 mg/L

The initial bacterial loads in control mice (0-h) for *S. pneumoniae* (R-1) were between 7.00 and 7.12 log_10_ cfu/lungs. The growth control showed an increase in bacterial counts of between 0.96 and 1.26 Δlog_10_ cfu/lungs after 24 h from the initiation of ceftriaxone therapy. The treatment with ceftriaxone significantly reduced the bacterial counts of *S. pneumoniae* (R-1) in the lungs compared with those in the control animals at 24 h (1 g twice-daily regimen, −0.42 ± 0.37 Δlog_10_ cfu/lungs, *P* < 0.001; 2 g once-daily regimen, −0.33 ± 0.18 Δlog_10_ cfu/lungs, *P* < 0.001). However, contrary to the results for *S. pneumoniae* with MICs of 1 and 2 mg/L, the antimicrobial activity of the 2 g once-daily regimen against *S. pneumoniae* with an MIC of 4 mg/L was similar to that of the 1 g twice-daily regimen (*P* = 0.684).

## Discussion

This study evaluated the antimicrobial activities of a ceftriaxone dosing regimen—administered as either 1 g twice-daily or 2 g once-daily—against S. *pneumoniae* isolates with various MICs utilizing a murine pneumonia model. The 2 g once-daily regimen exhibited superior antimicrobial activities against *S. pneumoniae* isolates with MICs of ≤2 mg/L compared with the 1 g twice-daily regimen. Meanwhile, the antimicrobial activities against S. *pneumoniae* isolate with an MIC of 4 mg/L were similar between the two regimens.

Pharmacokinetics must be considered during the treatment of bacterial infections. The percentage of time spent above the MIC (%TAM) is an important characteristic of ceftriaxone,^13^ thereby indicating that ceftriaxone exerts its antimicrobial activity in a time-dependent manner. A previous study has demonstrated that ceftriaxone requires %TAM of >40% to achieve an 85%–100% bacteriologic cure rate.^14^ It has been reported that the 2 g once-daily regimen shows %TAM of 100% in clinical isolates, due to the longer serum half-life among cephalosporins (8 h) in clinical cases.^15^ On the other hand, the 1 g twice-daily regimen shows %TAM of 69.3% against clinical isolates, whereas %TAM may drop from 40% in clinical isolates with further low susceptibility against ceftriaxone.^16^ In the time-kill assay using *Staphylococcus aureus* isolates with ceftriaxone MIC of 2 and 4 mg/L, the 1 g twice-daily regimen did not decrease the bacterial burden over the first 24 h of therapy,^17^ while the 2 g once-daily regimen achieved an approximate 1-log_10_ reduction in the bacterial burden over 24 h.^17,18^ These studies have demonstrated that the 2 g once-daily regimen exhibits greater antimicrobial activity than the 1 g twice-daily regimen. In other words, these results are akin to our *in vivo* study that assessed ceftriaxone 2 g once- and 1 g twice-daily regimens’ results against the *S. pneumoniae* isolates with MICs of 1 and 2 mg/L. Therefore, achieving a high ceftriaxone concentration in the early stages may be advantageous in improving the likelihood of achieving the desired bacterial killing target. In fact, our previous study showed that the 2 g once-daily regimen reduced inflammatory markers earlier than the 1 g twice-daily regimen.^8^ The clinical efficacy of the 2 g once-daily regimen is corroborated by the results obtained in this study.

Frequently, the blood concentration of antimicrobials is linked to their antimicrobial activities.^19^ Similarly, the concentration of antimicrobials within the tissue of target organs plays a critical role in determining their clinical efficacy.^20^ In our study, the duration for which concentrations exceeded the MIC of 4 mg/L in lung tissues of mice administered ceftriaxone at doses of 200, 400 and 800 mg/kg were 1.75, 4 and 8 h, respectively (Figure 1b). Although concentrations of ceftriaxone in lung tissues of mice receiving 236.15 and 520.85 mg/kg (corresponding to 1 and 2 g dose regimens, respectively) were not measured, the percentage of time above the MIC (%TAM) for the 2 g once-daily regimen is likely to exceed that of the 1 g twice-daily regimen. Consequently, sustaining high concentrations of ceftriaxone in lung tissue may improve its antimicrobial efficacy.

In the initial stages of infection, the production of proinflammatory cytokines is initiated by bacterial products.^21,22^ In cases of pneumonia, cytokines are generated in the bronchoalveolar lavage fluid and lung tissue, correlating with the severity of CAP.^23,24^ An *in vivo* study utilizing a murine pneumonia model has further demonstrated a significant release of cytokines at the infection site within 24 h of infection.^25^ Thus, reducing cytokine production within this timeframe could lead to improved clinical outcomes. Antimicrobials exhibit immunomodulatory effects that influence cytokine production, potentially lowering cytokine levels.^26^ A prior clinical study revealed that ceftriaxone decreased concentrations of cytokines (IL-1β, IL-6 and IL-8) starting from 24 h after the initiation of ceftriaxone therapy in patients with pneumococcal pneumonia, an effect sustained up to 120 h.^22^ Another study indicated a dose-dependent effect.^27^ Consequently, administering a high dose of ceftriaxone in the early stages of infection may amplify its immunomodulatory effects. Therefore, it is advisable not to split the daily dose of ceftriaxone.

This study identified an optimal dosing regimen of 2 g daily for ceftriaxone. Nevertheless, there are several limitations to this study. Firstly, only three clinical isolates of *S. pneumoniae* were examined, which may restrict the applicability of the findings to other isolates with varying MICs. However, the MIC cut-off based on susceptibility, as provided by the CLSI, was employed. Secondly, changes in bacterial density in lung tissue might be influenced by the inherent proliferative capacity and viability of each isolate. Thirdly, it was not possible to conduct protein binding studies; only total concentrations of ceftriaxone were measured in blood samples and lung tissues. Lastly, we did not assess the concentration of proinflammatory cytokines in the lung, indicating that further research into cytokine production in the lung is required to elucidate the optimal dosing regimen for ceftriaxone.

In conclusion, this study demonstrated that a human-simulated 2 g once-daily regimen of ceftriaxone was effective in a murine model of pneumonia caused by *S. pneumoniae* with a MIC of ≤2 mg/L, reducing bacterial density in lung tissue. Furthermore, the antimicrobial activity of ceftriaxone may contribute to the improvement of clinical symptoms in the early stages of infection. Therefore, a 2 g once daily regimen appears to be a favorable dosing regimen for patients with aspiration pneumonia caused by *S. pneumoniae,* with an MIC of ≤2 mg/L. The use of a local antimicrogram could be crucial for the empirical treatment of patients with pneumonia caused by *S. pneumoniae*.

## Data Availability

All data relevant to this study are available within the paper.
